# Prognostic Value of P63 Expression in Muscle-Invasive Bladder Cancer and Association with Molecular Subtypes—Preliminary Report

**DOI:** 10.3390/cimb46030155

**Published:** 2024-03-14

**Authors:** Francesca Sanguedolce, Ugo Giovanni Falagario, Magda Zanelli, Andrea Palicelli, Maurizio Zizzo, Stefano Ascani, Simona Tortorella, Gian Maria Busetto, Angelo Cormio, Giuseppe Carrieri, Luigi Cormio

**Affiliations:** 1 Pathology Unit, Policlinico Foggia, University of Foggia, 71122 Foggia, Italy; francesca.sanguedolce@unifg.it (F.S.); simona.tortorella@unifg.it (S.T.); 2 Department of Urology and Renal Transplantation, Policlinico Foggia, University of Foggia, 71122 Foggia, Italy; ugofalagario@gmail.com (U.G.F.); gianmaria.busetto@unifg.it (G.M.B.); giuseppe.carrieri@unifg.it (G.C.); luigi.cormio@unifg.it (L.C.); 3 Pathology Unit, Azienda USL-IRCCS di Reggio Emilia, 42123 Reggio Emilia, Italy; magda.zanelli@ausl.re.it (M.Z.); andrea.palicelli@ausl.re.it (A.P.); 4 Surgical Oncology Unit, Azienda USL-IRCCS di Reggio Emilia, 42123 Reggio Emilia, Italy; maurizio.zizzo@ausl.re.it; 5 Pathology Unit, Azienda Ospedaliera Santa Maria di Terni, University of Perugia, 05100 Terni, Italy; s.ascani@aospterni.it; 6 Urology Unit, Azienda Ospedaliero-Universitaria Ospedali Riuniti Di Ancona, Università Politecnica Delle Marche, 60126 Ancona, Italy; 7 Department of Urology, Bonomo Teaching Hospital, 76123 Andria, Italy

**Keywords:** muscle-invasive bladder cancer, P63, prognosis, molecular subtyping, radical cystectomy

## Abstract

There is an ongoing need for biomarkers that could reliably predict the outcome of BC and that could guide the management of this disease. In this setting, we aimed to explore the prognostic value of the transcription factor P63 in patients with muscle-invasive bladder cancer (MIBC) having undergone radical cystectomy. The correlation between P63 expression and clinicopathological features (tumor stage, nodes involvement, patterns of muscularis propria invasion, papillary architecture, anaplasia, concomitant carcinoma in situ, lymphovascular invasion, perineural invasion, necrosis) and molecular subtyping (basal and luminal type tumors) was tested in 65 radical cystectomy specimens and matched with cancer-specific survival (CSS) and overall survival (OS). P63-negative tumors displayed significantly higher rates of pattern 2 of muscularis propria invasion (50% vs. 14%, *p* = 0.002) and variant histology (45% vs. 19%, *p* = 0.022) compared to P63-positive ones. According to the combined expression of CK5/6 and CK20 (Algorithm #1), P63-positive and P63-negative tumors were mostly basal-like and double-negative, respectively (*p* = 0.004). Using Algorithm #2, based on the combined expression of CK5/6 and GATA3, the vast majority of tumors were luminal overall and in each group (*p* = 0.003). There was no significant difference in CSS and OS between P63-positive and P63-negative tumors, but the former featured a trend towards longer OS. Though associated with pathological features harboring negative prognostic potential, P63 status as such failed to predict CSS and OS. That said, it may contribute to better molecular subtyping of MIBC.

## 1. Introduction

Bladder cancer (BC) is the 7th most common malignancy worldwide, with a 5-year estimated number of prevalent cases as high as 1,720,625 worldwide, in both sexes [1]. Approximately 75% of BC patients present with non-muscle-invasive disease (NMIBC), which can often be cured with non-invasive treatments such as local resection, intravesical instillations, and stringent follow-up [2] and portends better clinical outcomes in terms of longer survival and lower rates of cancer-specific mortality in comparison with higher-stage tumors [3]. However, up to 20% of NMIBCs progress to muscle-invasive BC (MIBC) [4], which in turn is amenable for local (surgery, radiotherapy) and/or systemic therapy (chemotherapy, immunotherapy). According to current guidelines, risk stratification models rely on clinical and pathological parameters [2], with suboptimal results, highlighting the need for incorporating tissue biomarkers in order to reflect the biological heterogeneity of this disease and refine the patients’ prognosis [5].

In the last decade, several studies based on transcriptional analysis have resulted in different attempts at molecular subtyping of MIBCs, all of them sharing the top-level distinction into luminal and basal tumor types, as highlighted by a recent consensus classification [6,7], with each subtype being associated with peculiar clinical features as well as variable sensitivity to treatments. Immunohistochemical (IHC) antibodies have been described as effective surrogate markers for both luminal (CK20, GATA3) and basal (high-molecular-weight cytokeratins, such as CK5/6 and CK14) tumors, in order to translate research findings into routine clinical practice [8,9].

P63 is a transcription factor belonging to the P53 family, which comprises also P73. The paralog nature of these molecules results in any of them to variably influence each other, and it has been recently pointed out that P53 and P63 might collaborate, at least in some contexts, rather than compete [10]. In detail, P63 is involved in both epidermal development and the maintenance of cells of mesenchymal and germinal origin [11]. Its transcript variant ΔNp63 can be found in selected epithelia from different organs, including urothelial basal and intermediate cells, as well as basal cells in the breast and prostate. Moreover, tumors arising from these cells may contain a high amount of ΔNp63, which is able to support cell growth and hamper their differentiation [11].

Variable P63 expression rates have been seen in association with different BC stages, grades, and clinical outcomes, yet the prognostic value of P63 for MIBC remains controversial. This study aimed to explore the prognostic role of P63 in a cohort of MIBCs, by assessing its association with clinicopathological variables and molecular subtypes.

## 2. Materials and Methods

### 2.1. Study Cohort

This study focused on 65 patients having undergone radical cystectomy (RC) for MIBC at Foggia University Hospital between 2015 and 2021, for whom qualitatively and quantitatively optimal formalin-fixed paraffin-embedded tumor tissue was available for IHC analysis. In order to achieve a clinically homogeneous cohort, exclusion criteria were: (i) having received neoadjuvant or adjuvant chemotherapy, (ii) evidence of distant metastasis. Fifty patients were excluded on the basis of these criteria.

Hematoxylin–eosin and IHC slides from all tumor specimens were evaluated by two dedicated uropathologists (F.S. and S.T.). The following clinical and pathological variables were retrospectively gathered for each patient: age, gender, tumor stage (pT) according to the latest TNM staging system [12], pelvic nodes involvement, patterns of muscularis propria invasion classified as 1 (non-infiltrative, namely trabecular/nodular) and 2 (infiltrative) according to Haghayeghi et al. [13], histotype (conventional transitional cell carcinoma vs. variant histology), papillary architecture, anaplasia, concomitant carcinoma in situ, lymphovascular invasion, perineural invasion, and necrosis. All parameters were available for each patient.

### 2.2. Immunohistochemical Procedures

IHC staining was performed as previously described [14]. In brief, serial sections 4 μm thick were cut from formalin-fixed paraffin-embedded selected blocks, deparaffinized in xylene, rehydrated in graded ethanol solutions, washed for 5 min with distilled water, and mounted on poly-l-lysine-coated glass slides.

IHC staining was obtained by standard linked streptavidin–biotin horseradish peroxidase technique using specific monoclonal antibodies against P63 (mouse monoclonal primary antibody, clone 4A4, #05867061001), CK5/6 (mouse monoclonal primary antibody, clone D5/16B4, #06478441001), CK20 (rabbit monoclonal primary antibody, clone SP33, #05587760001), and GATA3 (mouse monoclonal primary antibody, clone L50-823, #07107749001), delivered by the Benchmark XT autostainer (Ventana Medical Systems Inc., Tucson, AZ, USA). All antibodies were supplied by Roche-Ventana. Positive and negative controls were used. Subsequently, the expression of every single antibody was evaluated under light microscopy throughout the whole section by two independent observers (F.S. and S.T.), and consensus was reached in each case. Nuclear expression of GATA3 and P63 and membranous/cytoplasmic expression of CK5/6, CK20 were assessed using the H-score according to the following formula: 0x% of cells with staining ‘0′ + 1x% staining ‘1′ + 2x% staining ‘2′ + 3x% staining ‘3′. Cases were stratified as negative/positive on the basis of an optimized cut-off of 150. Finally, all tumors were classified into 4 molecular subtypes: luminal (Lum), basal (Bas), double-positive (DP), and double-negative (DN), according to the expression of the following surrogate IHC markers: CK5/6 and CK20 [15], and CK5/6 and GATA3 [16], which from here on will be defined as “Algorithm #1” and “Algorithm #2”, respectively.

### 2.3. Statistical Analysis

Continuous variables are reported as median and interquartile range and tested by the Mann–Whitney U test, whereas categorical variables are reported as rates and tested by the Fisher’s exact test or the chi-square test, as appropriate. Overall survival (OS) and cancer-specific survival (CSS) were estimated nonparametrically using the Kaplan–Meier method, with differences among groups being tested for significance using the Log-rank test. The analyses were performed using Stata-SE 14 (StataCorp LP, College Station, TX, USA), and all tests were 2-sided with the significance level set at *p* < 0.05.

## 3. Results

### 3.1. Patients Clinicopathological Characteristics

**Figure 1:**
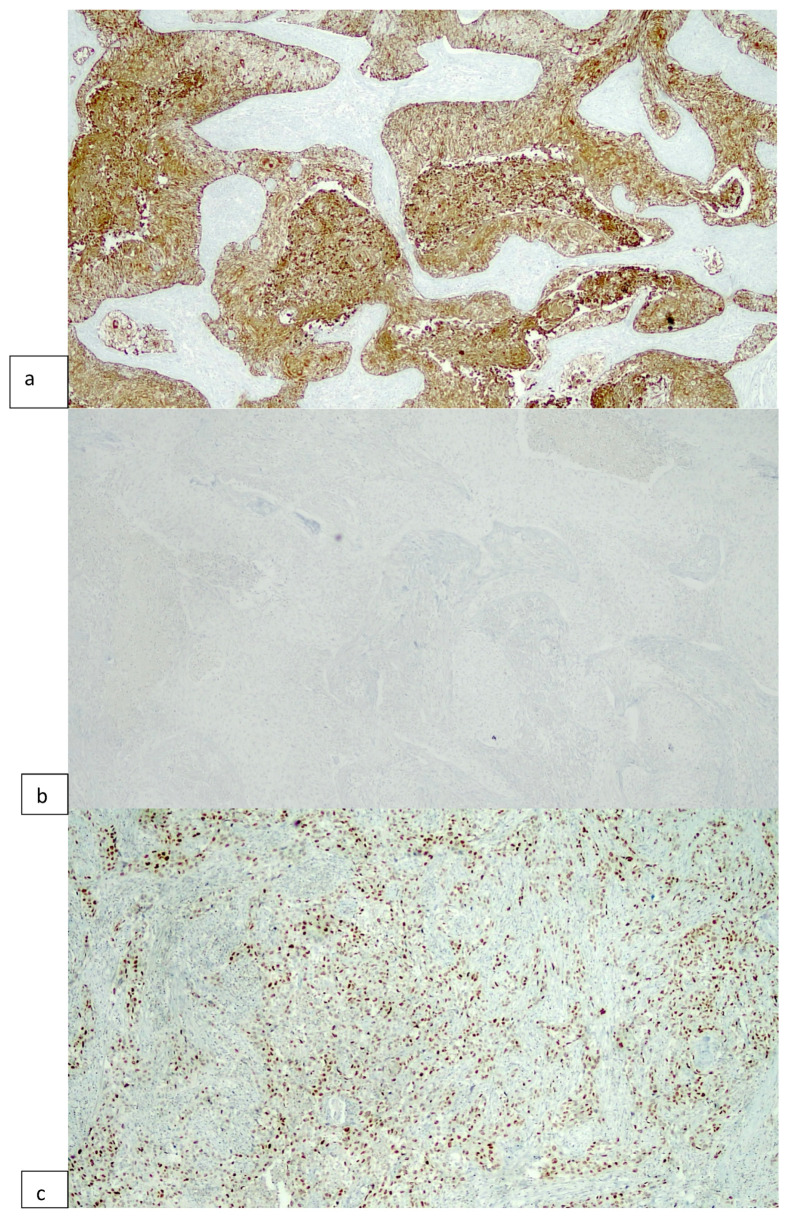
A basal tumor according to Algorithm #1, showing strong CK5/6 staining (**a**) and lacking CK20 expression (**b**). P63 is diffusely positive in tumor cells (**c**) (original magnification 100×).

Table 1 shows the clinicopathological features of the 2 sample groups, namely P63-negative (n. 22) and P63-positive (n. 43) patients. (Figure 1). The groups were similar in terms of age and gender, with male gender being overall more common. There was no difference in tumor stage and presence of pelvic nodes involvement. Conversely, pattern 2 of muscularis propria invasion was significantly more common in P63-negative patients (50% vs. 14%, *p* = 0.002). Variant histology was significantly more common in P63-negative patients (45% vs. 19%, *p* = 0.022). Specifically, P63-positive tumors included 6 squamous, 1 sarcomatoid and 1 nested variant, whereas P63-negative tumors included 7 micropapillary and 3 plasmocitoid tumors.

Most tumors were non-papillary and lacked anaplasia, again with no difference among the two groups. Concomitant CIS was more common in P63-negative tumors, but the difference did not reach statistical significance (36% vs. 16%, *p* = 0.069). LVI, PNI, and necrosis were similar in the two groups.

### 3.2. P63 Expression and Molecular Subtyping

Table 2 shows the distribution of the 2 groups of P63-negative and P63-positive tumors across the 4 subtypes according to the combined expression of 2 luminal (CK20, GATA3) and 1 basal marker (CK5/6) within the 2 proposed algorithms.

According to Algorithms #1 and #2, P63-negative tumors are mostly CK5/6-/CK20- (DN) and CK5/6-/GATA3+ (Lum) (12/22, 57%, vs.18/22, 83%, respectively), whereas P63-positive MIBCs were most commonly enriched in the CK5/6+/CK20- (Bas) and CK5/6-/GATA3+ (Lum) clusters (17/43, 40%, vs. 25/43, 58%, respectively). Luminal MIBCs were overrepresented in both classification systems (23/65, 35% and 43/65, 66%, respectively), followed, in decreasing order, by DN, Bas, and DP tumors (Algorithm #1), and DP, Bas, and DN tumors (Algorithm #2), respectively. Differences among groups were statistically significant.

### 3.3. Survival Analysis

**Figure 2:**
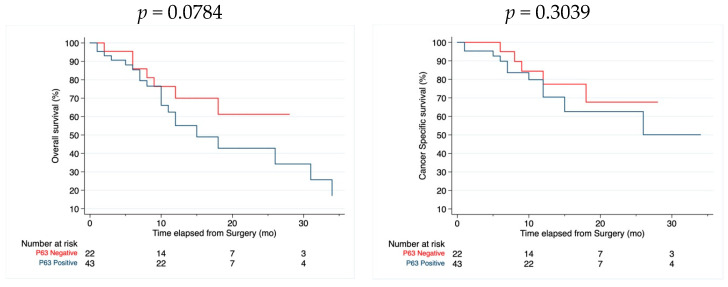
Overall and cancer-specific Kaplan–Meier curves.

There was no difference in CSS and OS between P63-negative and P63-positive tumors (Figure 2), but P63-negative tumors featured a trend towards better OS then the P63-positive ones (*p* = 0.0784).

## 4. Discussion

We aimed to explore the prognostic role of P63 in a homogeneous cohort of MIBC patients treated with RC, by assessing its association with clinicopathological variables and molecular subtypes. We found that P63 status was significantly associated with morphological features which are known to carry a negative prognostic potential, namely the presence of variant histology and pattern 2 of muscularis propria invasion.

Due to its high sensitivity, P63 is recommended as part of a multi-antibody panel (usually along with GATA3, uroplakin II, and high-molecular-weight cytokeratin) in order to establish the urothelial lineage and rule out a secondary tumor to the bladder [17]. In this setting, its low specificity should be taken into account, especially when the differential diagnosis involves prostate cancer, since P63 can be aberrantly expressed by a minor subset (less than 5%) of such tumors [18].

Within urothelial lesions of the bladder, P63 has shown consistently homogeneous expression between primary and nodal metastases, with a concordance rate as high as 75% [19], in disagreement with a previous study, which reported on differential rates of P63 protein and mRNA levels in primary tumors as compared to their matched nodal metastases [20]. A study addressing intratumoral heterogeneity through IHC in BC tissue microarrays described low rates of divergent P63 staining among different cores from the same tumors (81/948, 9%) [21]. In our study, heterogeneity was not seen when reviewing representative full slides of each case. It is worth mentioning that the presence of two major protein variants with well-defined expression patterns and functional features, namely Tap63 and ΔNp63, may affect IHC identification of P63 in both normal and neoplastic urothelial cells. Monoclonal antibodies specific for Tap63 do not stain normal adult urothelial nuclei, whereas the opposite occurs when using antibodies targeting ΔNp63 [12]. The most used antibody clone is 4A4, which is raised against the N-terminus of ∆Np63 isoform; hence, from here on, with regards to IHC, we will refer to this specific clone unless otherwise specified.

The present study demonstrated that, in patients with MIBC having undergone RC, P63 status was significantly associated with two morphological features known to carry a negative prognostic potential, namely variant histology and pattern 2 of muscularis propria invasion.

According to Compérat et al., consistently different P63 expression rates can be detected in low- vs. high-grade, as well as low- vs. high-stage bladder tumors, with lower-grade and -stage tumors showing more intense and homogeneous staining [22], as confirmed by a later study [23]. Such findings support the role of P63 assessment in the differential diagnosis of low- and high-grade NMIBCs, along with its gradual decrease over disease progression. Accordingly, the deregulation of P63 has been reported as an early event during bladder carcinogenesis in an analysis performed at both RNA and protein levels, which also disclosed a close correlation between reverse transcriptase–polymerase chain reaction (RT–PCR) and IHC results, thus suggesting the latter as a feasible and reliable method to investigate P63 status in tissue samples [24].

A recent study supported the adverse prognostic role of P63, along with other epithelial–mesenchymal transition (EMT) markers, such as β-catenin and E-cadherin, in urothelial carcinomas [25]. EMT has been defined as a cellular reprogramming mechanism leading to a more aggressive, potentially metastatic tumor type [26]. ∆Np63 is known as an EMT regulator through multiple miRNA and cell signaling pathways, encompassing PTEN/AKT, TGFβ, Wnt, and Notch [27]. These findings further support the hypothesis that P63 can be used as a marker to identify a more aggressive subset of MIBCs. Indeed, Koga et al. found a statistically significant association between lower P63 expression and higher-stage (*p* = 0.0004), nodal metastases (*p* = 0.003), and poor prognosis (*p* = 0.0005) [28]. Our study failed to demonstrate an association between P63 expression and disease stage or nodal metastases. Conversely, we found that variant histology was significantly more common in P63-negative tumors; moreover, the more aggressive histotypes (plasmocitoid and micropapillary) were much more frequent in P63-negative tumors, whereas the sarcomatoid type was more frequent in P63-positive tumors. P63 expression is known to vary across BCs with variant histology [29], being lower to absent in micropapillary and plasmacytoid carcinomas, and higher in BC with squamous differentiation, nested and sarcomatoid carcinoma. The negative prognostic value of variant histology is well known, particularly for micropapillary and plasmacytoid types, to the extent that its occurrence should be included in the pathology report, even if present as a minor component within an otherwise conventional urothelial carcinoma [30]. Another morphological finding of our study was pattern 2 of MP invasion being significantly more common in P63-negative tumors. In a large French multicenter trial, this was found to be a significant predictor of tumor recurrence and progression as well as of worse CSS [31]. In spite of these morphological findings, our study failed to demonstrate an association between P63 status and CSS or OS, but there was a trend towards worse OS in P63-positive tumors. This issue is controversial. Choi et al. reported that P63-positive MIBCs had significantly shorter OS (*p* = 0.07) [32]. Similarly, Wang et al. [33] found that P63 overexpression, along with advanced-stage parameters (T and N), ABO blood type, and a history of diabetes mellitus, functioned as an independent factor for worse survival in a cohort of 103 MIBCs treated with RC. Burgess et al. failed to find a significant association between P63 status and clinical outcome [34]. Conversely, a recent large tissue microarray study on 10,200 tumors described a consistent association with advanced stage, high grade, and poor survival (*p* < 0.0001) in P63-negative UCs [22]. In line with these findings, Koga et al. reported a significantly shorter 5-year CSS in the low vs. high P63 expression group [28].

One potential reason for such divergent findings across previous studies may be due to the fact they included tumors of different stage and grade as well as to large variability in analytical parameters, specifically different antibodies and staining protocols [35], different cut-offs of stained cells (10%, 50%, 80%), combined evaluation of staining intensity, and percentage of positive cells (Table 3). To minimize such potentially confusing factors, we chose to analyze a homogeneous cohort of MIBCs treated with RC only.

Since there is no current standardized scoring system for P63, we used the H-score, a type of immunoreactivity score which appoints an ordinal value to the staining intensity and multiplies this by the percentage of positive cells, resulting in a range of 0–300 final scores [36]. The H-score is widely used mostly in the research setting, to assess group-specific differences [36]. Moreover, we used two double-antibody panels as surrogate markers in order to stratify MIBCs according to current molecular subtyping systems. This is relevant in view of the fact that the recent introduction of a consensus molecular classification of MIBC [7] resulted in the attempt to implement such data in clinical practice through the development of a limited antibody panel. In the Lund University classification system, urobasal tumors showed high expression of P63 along with FGFR3 and CCND1, and such tumors were found to be associated with more favorable disease-specific survival; conversely, genomically unstable (GU) tumors were mostly P63-negative [37]. However, the urobasal group encompassed the highest percentage of NMIBCs (86%) as compared to the other two subtypes, namely squamous cell cancer-like (SCCL) and GU tumors (12% and 65%, respectively). In view of the similarities between the luminal/basal dichotomy of breast cancers and a BC subtyping system proposed by Choi et al. [38], P63 has been commonly regarded as a basal marker, in that it induces a basal phenotype with certain homeostatic signaling pathways; conversely, the peroxisome proliferator activator receptor (PPAR) gamma γ (PPARγ) is a known luminal marker and drives different transcription factors. Basal and luminal phenotypes are enriched in several different genetic alterations and pathways, the former being reportedly associated with more EMT and stem cell markers as well as squamous and sarcomatoid histological features that are known to portend worse outcomes [6]. Nevertheless, the use of P63 as a basal marker for molecular subtyping has been challenged due to its wide expression in urothelial carcinomas [39].

Previously, we performed a comprehensive two-part literature review on the available immunohistochemical markers to be implemented as surrogates in an attempt to stratify MIBCs according to current molecular subtyping systems [8,9]. As a consequence, we chose two double-antibody panels which proved to properly distinguish between different classes of tumors [15,16]. Interestingly, P63-positive and P63-negative tumors were mostly basal and double-negative, respectively (*p* = 0.004), according to the combined expression of CK5/6 and CK20 (Algorithm #1). Conversely, using Algorithm #2, the vast majority of tumors were luminal overall, as well as in each group (*p* = 0.003). This discrepancy may be explained by the differential use of CK20 and GATA3 as luminal markers in the two proposed algorithms, since these antibodies have distinct sensitivity rates. CK20 has been reported to stain 29–89% of BCs overall [40], whereas GATA3 diffuse, nuclear positivity has been described in >85% of UCs, irrespective of grade [18]. On the basis of our findings, and of the available literature [7,8], we suggest the combined use of both GATA3 and CK20 as luminal markers in routine practice. Since luminal and basal subtypes portend different clinical outcomes, acknowledging the characteristics of each biomarker as well as how to properly combine them is of pivotal importance.

The predictive potential of P63 has been also explored by some authors, who suggested that its expression may enhance the sensitivity of BC cells to the antimitotic agent AZD4877 on the basis of an in vitro study [41], highlighting that those patients with P63-positive MIBC could benefit from treatment with antimitotic chemotherapy. In their recent comprehensive review, Pokorna et al. [11] enlisted a series of therapeutic strategies that, by targeting P63 in high-expressing tumors, may obtain long-term inhibition of malignant cell proliferation. They include (1) agents inhibiting cell signaling pathways (such as EGFR and PI3K/mTOR), resulting in the downregulation of ΔNp63; (2) conventional chemotherapy with cisplatin and other genotoxic agents, which induce activation of Tap63 and disruption of ΔNp63, furthermore, ΔNp63 levels have been reported to confer cisplatin sensitivity to subsets of squamous cell carcinomas and breast cancers [42,43]; and (3) the use of targeted ultraviolet light, to degrade P63 in anatomically accessible urothelial cancers.

Our study has some potential limitations. First, it is a retrospective study on a limited population; however, it is a well-selected and homogeneous cohort of MIBCs. We studied patients for whom follow-up data were available and selected them based on the inclusion and exclusion criteria mentioned above. To overcome this limitation, and achieve more significant results, we are planning to expand our cohort in a future study. Second, the presence of a relatively high rate of variant histology in our series may represent an inherent bias, yet it allows us to draw meaningful results. Finally, the predictive potential of P63 has not been assessed since no treatments other than RC were given.

## 5. Conclusions

This is the first study assessing the prognostic role of p63 in relation to molecular subtyping of BC; hence, although these are preliminary findings, it is one of a kind among research on prognostic markers in BC. There is a strong need for biomarkers that could reliably predict the outcome of BC and that could guide the management of this disease. The present study demonstrated that, in patients with MIBC having undergone RC, P63 status was significantly associated with morphological features which are known to carry a negative prognostic potential, namely the presence of variant histology and pattern 2 of muscularis propria invasion. In spite of such findings, P63 status as such failed to predict CSS and OS, even though there was a trend towards a lower overall survival for P63-positive tumors. Finally, when associated with other surrogate IHC markers, P63 status may contribute to better molecular subtyping of MIBCs, but further research is needed to define the best algorithm and to assess the prognostic and the predictive values of such findings.

## Figures and Tables

**Table 1:** Descriptive characteristics of the study population.

	P63-Negative (*n* = 22)	P63-Positive (*n* = 43)	*p* Value
**Age, mean (range)**	71 (68–78)	76 (70–82)	0.077
**Gender, *n* (%)**			
Female	3 (14%)	5 (12%)	0.8
Male	19 (86%)	38 (88%)	
**Papillary architecture, *n* (%)**			
Absent	20 (91%)	36 (84%)	0.4
Present	2 (9%)	7 (16%)	
**DD/HS, *n* (%)**			
Absent	12 (55%)	35 (81%)	0.022
Present	10 (45%)	8 (19%)	
**Anaplasia, *n* (%)**			
Absent	20 (91%)	35 (81%)	0.3
Present	2 (9%)	8 (19%)	
**CIS, *n* (%)**			
Absent	14 (64%)	36 (84%)	0.069
Present	8 (36%)	7 (16%)	
**pT, *n* (%)**			
2	4 (18%)	7 (16%)	0.8
3	11 (50%)	25 (58%)	
4	7 (32%)	11 (26%)	
**Pelvic nodal involvement, *n* (%)**			
Absent	12 (55%)	21 (49%)	0.7
Present	10 (45%)	22 (51%)	
**LVI, *n* (%)**			
Absent	9 (41%)	23 (53%)	0.3
Present	13 (59%)	20 (47%)	
**PNI, *n* (%)**			
Absent	16 (73%)	24 (56%)	0.2
Present	6 (27%)	19 (44%)	
**Pattern of MP invasion, *n* (%)**			
1	11 (50%)	37 (86%)	0.002
2	11 (50%)	6 (14%)	
**Necrosis, *n* (%)**			
Absent	9 (41%)	16 (37%)	0.8
Present	13 (59%)	27 (63%)	

CIS: Carcinoma In Situ; DD: Divergent Differentiation; HS: Histological Subtype; LVI: Lymphovascular Invasion; MP: Muscularis Propria; PNI: Perineural Invasion.

**Table 2:** Association of P63 expression with molecular subtyping algorithms.

	P63-Negative (*n* = 22)	P63-Positive (*n* = 43)	*p* Value
**Algorithm #1, *n* (%)**			
Bas	0 (0%)	17 (40%)	0.004
Lum	8 (38%)	15 (35%)	
DP	1 (5%)	1 (2%)	
DN	12 (57%)	10 (23%)	
**Algorithm #2, *n* (%)**			
Bas	0 (0%)	5 (12%)	0.003
Lum	18 (82%)	25 (58%)	
DP	1 (5%)	13 (30%)	
DN	3 (14%)	0 (0%)	

Bas: basal; DN: double-negative; DP: double-positive; Lum: luminal.

**Table 3:** P63 as a diagnostic and prognostic biomarker in bladder lesions: findings from selected studies.

Grade	Stage	Treatment	Clone	Target	Scoring Method	Findings	Reference
HG	MIBCs	NAC + RC	4A4	∆Np63 isoform	Overexpression > 50% stained cells	Baseline p63 (HR 2.02; 95% CI = 0.51–8.1; *p* = 0.313) protein expression did not predict for OS.	[34]
LG + HG	NMIBCs + MIBCs	NA	DAK-p63	TAp63 and ΔNp63 isoforms	Low (≤median), high (>median)	Higher P63 expression showed a significant association with low-grade tumors (*p* < 0.05).	[23]
HG	NMIBCs + MIBCs	RC	4A4	∆Np63 isoform	Combined intensity and percentage of stained cells	Lower P63 expression showed a significant association with higher-stage (*p* = 0.0004), nodal metastases (*p* = 0.0013), and poor prognosis (*p* = 0.0005).	[28]
LG + HG	NMIBCs + MIBCs	RC	4A4	∆Np63 isoform	Negative (<10%), weak (10–80%), high (80–100%) stained cells	Higher P63 expression showed a significant association with poor OS (*p* < 0.001) in patients with MIBC.	[25]
LG + HG	NMIBCs + MIBCs	RC	NA	NA	0 (≤10%), + (>10%) stained cells	Positive P63 expression was an independent factor for worse survival (*p* = 0.033) in all patients.	[33]
LG + HG	NMIBCs + MIBCs	TUR, RC	4A4	∆Np63 isoform	0 (<10%), 1 (10–80%), 2 (80–100%) stained cells	P63 expression distinguished between PUNLMP/NILGC and NIHGC/pT1 (*p* = 4.10^5^).	[36]

HG: high grade; LG: low grade; MIBC: muscle-invasive bladder cancer; NA: not available; NAC: neoadjuvant chemotherapy; NIHGC: non-invasive high-grade carcinoma; NILGC: non-invasive low-grade carcinoma; NMIBC: non-muscle-invasive bladder cancer; OS: overall survival; PUNLMP: papillary urothelial neoplasm of low malignant potential; RC: radical cystectomy; TUR: transurethral resection.

## Data Availability

The data presented in this study are available upon request from the corresponding author.

## References

[1] https://gco.iarc.fr/today/.

[2] Babjuk M., Burger M., Compérat E., Gontero P., Liedberg F., Masson-Lecomte A., Mostafid A.H., Palou J., Van Rhijn B.W.G., Roupret M. (2022). EAU Guidelines on Non-Muscle-Invasive Bladder Cancer (TaT1 and CIS).

[3] Witjes J.A., Bruins H.M., Carrión A., Cathomas R., Compérat E.M., Efstathiou J.A., Kietkau R., Gakis G., Van der Heijden A.G., Lorch A. (2022). EAU Guidelines on Muscle-Invasive and Metastatic Bladder Cancer.

[4] Matulewicz R.S., Steinberg G.D. (2020). Non-muscle-invasive Bladder Cancer: Overview and Contemporary Treatment Landscape of Neoadjuvant Chemoablative Therapies. Rev. Urol..

[5] Sanguedolce F., Cormio A., Bufo P., Carrieri G., Cormio L. (2015). Molecular markers in bladder cancer: Novel research frontiers. Crit. Rev. Clin. Lab. Sci..

[6] Robertson A.G., Kim J., Al-Ahmadie H., Bellmunt J., Guo G., Cherniack A.D., Hinoue T., Laird P.W., Hoadley K.A., Akbani R. (2017). Comprehensive Molecular Characterization of Muscle-Invasive Bladder Cancer. Cell.

[7] Kamoun A., De Reyniès A., Allory Y., Sjödahl G., Robertson A.G., Seiler R., Hoadley K.A., Groeneveld C.S., Al-Ahmadie H., Choi W. (2020). A Consensus Molecular Classification of Muscle-invasive Bladder Cancer. Eur. Urol..

[8] Sanguedolce F., Zanelli M., Palicelli A., Ascani S., Zizzo M., Cocco G., Björnebo L., Lantz A., Landriscina M., Conteduca V. (2022). Are We Ready to Implement Molecular Subtyping of Bladder Cancer in Clinical Practice? Part 2: Subtypes and Divergent Differentiation. Int. J. Mol. Sci..

[9] Sanguedolce F., Zanelli M., Palicelli A., Ascani S., Zizzo M., Cocco G., Björnebo L., Lantz A., Landriscina M., Conteduca V. (2022). Are We Ready to Implement Molecular Subtyping of Bladder Cancer in Clinical Practice? Part 1: General Issues and Marker Expression. Int. J. Mol. Sci..

[10] Woodstock D.L., Sammons M.A., Fischer M. (2021). p63 and p53: Collaborative Partners or Dueling Rivals?. Front. Cell Dev. Biol..

[11] Pokorná Z., Vysloužil J., Hrabal V., Vojtěšek B., Coates P.J. (2021). The foggy world(s) of p63 isoform regulation in normal cells and cancer. J. Pathol..

[12] Amin M.B., Greene F.L., Edge S.B., Compton C.C., Gershenwald J.E., Brookland R.K., Meyer L., Gress D.M., Byrd D.R., Winchester D.P. (2017). The Eighth Edition AJCC Cancer Staging Manual: Continuing to build a bridge from a population-based to a more “personalized” approach to cancer staging. CA Cancer J. Clin..

[13] Haghayeghi K., Lu S., Matoso A., Schiff S.F., Mueller-Leonhard C., Amin A. (2021). Association of current molecular subtypes in urothelial carcinoma with patterns of muscularis propria invasion. Virchows Arch..

[14] Cormio L., Sanguedolce F., Cormio A., Massenio P., Pedicillo M.C., Cagiano S., Calò G., Pagliarulo V., Carrieri G., Bufo P. (2017). Human epidermal growth factor receptor 2 expression is more important than bacillus calmette guerin treatment in predicting the outcome of T1G3 bladder cancer. Oncotarget.

[15] Kim B., Jang I., Kim K., Jung M., Lee C., Park J.H., Kim Y.A., Moon K.C. (2021). Comprehensive Gene Expression Analyses of Immunohistochemically Defined Subgroups of Muscle-Invasive Urinary Bladder Urothelial Carcinoma. Int. J. Mol. Sci..

[16] Guo C.C., Bondaruk J., Yao H., Wang Z., Zhang L., Lee S., Lee J.G., Cogdell D., Zhang M., Yang G. (2020). Assessment of Luminal and Basal Phenotypes in Bladder Cancer. Sci. Rep..

[17] Zhai Q., Deng F.M., Zhou H., Guo C.C., Ro J.Y. (2021). Diagnostic Values of Immunohistochemistry in Bladder Cancer. Urinary Bladder Pathology.

[18] Sanguedolce F., Russo D., Mancini V., Selvaggio O., Calò B., Carrieri G., Cormio L. (2019). Morphological and Immunohistochemical Biomarkers in Distinguishing Prostate Carcinoma and Urothelial Carcinoma: A Comprehensive Review. Int. J. Surg. Pathol..

[19] Bontoux C., Rialland T., Cussenot O., Compérat E. (2021). A four-antibody immunohistochemical panel can distinguish clinico-pathological clusters of urothelial carcinoma and reveals high concordance between primary tumor and lymph node metastases. Virchows Arch..

[20] Sjödahl G., Eriksson P., Lövgren K., Marzouka N.A., Bernardo C., Nordentoft I., Dyrskjøt L., Liedberg F., Höglund M. (2018). Discordant molecular subtype classification in the basal-squamous subtype of bladder tumors and matched lymph-node metastases. Mod. Pathol..

[21] Jakobsson L., Chebil G., Marzouka N.A., Liedberg F., Sjödahl G. (2018). Low Frequency of Intratumor Heterogeneity in Bladder Cancer Tissue Microarrays. Bladder Cancer.

[22] Compérat E., Camparo P., Haus R., Chartier-Kastler E., Bart S., Delcourt A., Houlgatte A., François R., Capron F., Vieillefond A. (2006). Immunohistochemical expression of p63, p53 and MIB-1 in urinary bladder carcinoma. A tissue microarray study of 158 cases. Virchows Arch..

[23] Abdallah M.M., Wahbbah M.A., Selem M., Abdou A.G., Sultan S.M. (2021). Correlation between immunohistochemical expression of Ki-67and P63 and aggressiveness of urinary bladder urothelial carcinoma. J. Immunoass. Immunochem..

[24] Compérat E., Bièche I., Dargère D., Ferlicot S., Laurendeau I., Benoît G., Vieillefond A., Verret C., Vidaud M., Capron F. (2007). p63 gene expression study and early bladder carcinogenesis. Urology.

[25] Moussa R.A., Khalil E.Z.I., Ali A.I. (2019). Prognostic Role of Epithelial-Mesenchymal Transition Markers “E-Cadherin, β-Catenin, ZEB1, ZEB2 and p63” in Bladder Carcinoma. World J. Oncol..

[26] Stacy A.J., Craig M.P., Sakaram S., Kadakia M. (2017). ΔNp63α and microRNAs: Leveraging the epithelial-mesenchymal transition. Oncotarget.

[27] Tian Y.H., He Y.F., Tan J.S., Jiang Y., Xu Q., Cheng H.L. (2022). The DeltaN p63 Promotes EMT and Metastasis in Bladder Cancer by the PTEN/AKT Signalling Pathway. Evid.-Based Complement. Altern. Med..

[28] Koga F., Kawakami S., Fujii Y., Saito K., Ohtsuka Y., Iwai A., Ando N., Takizawa T., Kageyama Y., Kihara K. (2003). Impaired p63 expression associates with poor prognosis and uroplakin III expression in invasive urothelial carcinoma of the bladder. Clin Cancer Res..

[29] Paner G.P., Annaiah C., Gulmann C., Rao P., Ro J.Y., Hansel D.E., Shen S.S., Lopez-Beltran A., Aron M., Luthringer D.J. (2014). Immunohistochemical evaluation of novel and traditional markers associated with urothelial differentiation in a spectrum of variants of urothelial carcinoma of the urinary bladder. Hum. Pathol..

[30] Witjes J.A., Babjuk M., Bellmunt J., Bruins H.M., De Reijke T.M., De Santis M., Gillessen S., James N., Mclennan S., Palou J. (2020). EAU-ESMO Consensus Statements on the Management of Advanced and Variant Bladder Cancer—An international collaborative multistakeholder effort: Under the auspices of the EAU-ESMO Guidelines Committees. Eur. Urol..

[31] Rouprêt M., Seisen T., Compérat E., Larré S., Mazerolles C., Gobet F., Fetissof F., Fromont G., Safsaf A., d’Arcier B.F. (2013). Prognostic interest in discriminating muscularis mucosa invasion (T1a vs. T1b) in nonmuscle invasive bladder carcinoma: French national multicenter study with central pathology review. J. Urol..

[32] Choi W., Shah J.B., Tran M., Svatek R., Marquis L., Lee I.L., Yu D., Adam L., Wen S., Shen Y. (2012). p63 expression defines a lethal subset of muscle-invasive bladder cancers. PLoS ONE.

[33] Wang L., Zhou M., Feng C., Gao P., Ding G., Zhou Z., Jiang H., Wu Z., Ding Q. (2016). Prognostic value of Ki67 and p63 expressions in bladder cancer patients who underwent radical cystectomy. Int. Urol. Nephrol..

[34] Burgess E.F., Livasy C., Trufan S., Zhu J., O’Connor H.F., Hartman A., Clark P.E., Grigg C., Raghavan D. (2022). Clinical outcomes associated with expression of aurora kinase and p53 family members in muscle-invasive bladder cancer. Mol. Clin. Oncol..

[35] Steurer S., Riemann C., Büscheck F., Luebke A.M., Kluth M., Hube-Magg C., Hinsch A., Höflmayer D., Weidemann S., Fraune C. (2021). p63 expression in human tumors and normal tissues: A tissue microarray study on 10,200 tumors. Biomark. Res..

[36] Meyerholz D.K., Beck A.P. (2018). Principles and approaches for reproducible scoring of tissue stains in research. Lab. Investig..

[37] Sjödahl G., Lövgren K., Lauss M., Patschan O., Gudjonsson S., Chebil G., Aine M., Eriksson P., Månsson W., Lindgren D. (2013). Toward a molecular pathologic classification of urothelial carcinoma. Am. J. Pathol..

[38] Choi W., Porten S., Kim S., Willis D., Plimack E.R., Hoffman-Censits J., Roth B., Cheng T., Tran M., Lee I.L. (2014). Identification of distinct basal and luminal subtypes of muscle-invasive bladder cancer with different sensitivities to frontline chemotherapy. Cancer Cell..

[39] Weyerer V., Weisser R., Moskalev E.A., Haller F., Stoehr R., Eckstein M., Zinnall U., Gaisa N.T., Compérat E., Perren A. (2019). Distinct Genetic Alterations and Luminal Molecular Subtype in Nested Variant of Urothelial Carcinoma. Histopathology.

[40] Sanguedolce F., Russo D., Calò B., Cindolo L., Carrieri G., Cormio L. (2019). Diagnostic and prognostic roles of CK20 in the pathology of urothelial lesions. A systematic review. Pathol. Res. Pract..

[41] Marquis L., Tran M., Choi W., Lee I.L., Huszar D., Siefker-Radtke A., Dinney C., McConkey D.J. (2012). p63 expression correlates with sensitivity to the Eg5 inhibitor ZD4877 in bladder cancer cells. Cancer Biol. Ther..

[42] Zangen R., Ratovitski E., Sidransky D. (2005). ΔNp63α levels correlate with clinical tumor response to cisplatin. Cell Cycle.

[43] Rocca A., Viale G., Gelber R.D., Bottiglieri L., Gelber S., Pruneri G., Ghisini R., Balduzzi A., Pietri E., D’Alessandro C. (2008). Pathologic complete remission rate after cisplatin-based primary chemotherapy in breast cancer: Correlation with p63 expression. Cancer Chemother. Pharmacol..

